# A Stealthiness Evaluation of Main Chain Carboxybetaine Polymer Modified into Liposome

**DOI:** 10.3390/pharmaceutics16101271

**Published:** 2024-09-28

**Authors:** Mazaya Najmina, Shingo Kobayashi, Rena Shimazui, Haruka Takata, Mayuka Shibata, Kenta Ishibashi, Hiroshi Kamizawa, Akihiro Kishimura, Yoshihito Shiota, Daichi Ida, Taro Shimizu, Tatsuhiro Ishida, Yoshiki Katayama, Masaru Tanaka, Takeshi Mori

**Affiliations:** 1 Department of Applied Chemistry, Faculty of Engineering, Kyushu University, Motooka 819-0395, Japan; 2 Institute for Materials Chemistry and Engineering, Kyushu University, Motooka 819-0395, Japan; 3 Graduate School of Systems Life Sciences, Kyushu University, Motooka 819-0395, Japan; 4 Department of Pharmacokinetics and Biopharmaceutics, Institute of Biomedical Sciences, Tokushima University, Tokushima 770-8503, Japan; 5 Center for Future Chemistry, Kyushu University, Motooka 819-0395, Japan; 6 Department of Polymer Chemistry, Kyoto University, Ikenoura Gokasho 611-0011, Japan; 7 Center for Advanced Modalities and DDS, Osaka University, Yamadaoka 565-0871, Japan

**Keywords:** zwitterionic polymer, carboxybetaine, non-fouling polymer, liposome

## Abstract

**Background:** Acrylamide polymers with zwitterionic carboxybetaine (CB) side groups have attracted attention as stealth polymers that do not induce antibodies when conjugated to proteins. However, they induce antibodies when modified onto liposomes. We hypothesized that antibodies are produced against polymer backbones rather than CB side groups. **Objectives:** In this study, we designed and synthesized a polymer employing CB in its main chain, poly(*N*-acetic acid-*N*-methyl-propyleneimine) (PAMPI), and evaluated the blood retention of PAMPI-modified liposomes in mice. **Results:** The non-fouling nature of PAMPI-modified liposomes estimated from serum protein adsorption was found to be not inferior to PCB- and PEG-modified liposomes. However, to our surprise, the PAMPI-modified liposomes showed an instantaneous clearance less than 1 h post-injection, comparable to the naked liposomes. **Conclusions:** The extent of the blood retention of polymer-modified liposomes cannot be predicted by their susceptibility to serum protein adsorption and semi-flexible conformation.

## 1. Introduction

PEGylation, i.e., modification with polyethylene glycol (PEG), has been used for extending the blood retention of protein and nanomedicine due to its non-fouling nature toward nonspecific protein adsorption. However, since it has been proved that these PEG conjugates induce anti-PEG antibodies in rodents, beagle dogs, pigs, and monkeys, as well as humans [1,2,3,4,5,6], researchers have tried to find polymers that do not induce antibodies. Polycarboxybetaines (PCBs) with zwitterionic carboxybetaine side chains are gathering attention as promising candidates. Since carboxybetaine units have low charge density due to the intramolecular ionic interaction, PCBs have a non-fouling nature toward nonspecific protein adsorption [7]. Protein conjugates with PCB have been reported to show negligible antigenicity [8,9]. 

However, our group recently confirmed that PCB showed antigenicity when it was modified onto liposomes. Liposomes (100 nm) can crosslink several B-cell receptors, which are known to have 20–30 nm F_ab_ spacing [10], triggering the production of low-affinity IgM. This implies that the antigenicity of PCB, depending on the modified object, may be explained by the size of the object. Since PEG-modified particles with smaller sizes (typically less than 30 nm) have been reported to have reduced antigenicity [11], the negligible antigenicity of PCB-modified proteins may be due to their small size. Compared with PEG, the antigenicity of PCB would be less, but PCB’s antigenicity is not satisfactory to avoid the antibody induction toward liposomes. 

**Figure 1:**
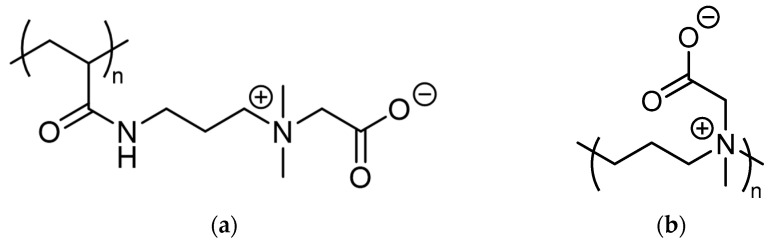
The chemical structures of (**a**) conventional PCB and (**b**) PAMPI including carboxybetaine in the main chain.

It has previously been reported that PCB is an acrylamide-based polymer (Figure 1a). This polymer includes amide and propyl groups, which are epitopes that induce antibodies via recognition through hydrogen bonding and hydrophobic interaction. Accordingly, even though the antigenicity of the CB group is weak, the amide and propyl groups may enhance the induction of antibodies to PCB. In this paper, therefore, we attempted to design a new PCB composed of minimal groups, except for carboxybetaine. Poly(*N*-acetic acid-*N*-methyl-propyleneimine) (PAMPI) is the newly designed PCB that contains carboxybetaine in its main chain (Figure 1b). We then examined the blood retention and antigenicity of the liposome modified with PAMPI.

## 2. Materials and Methods

### 2.1. Materials

2-Ethyl-2-oxazine, ethyl 3-bromopropionate, formic acid (85%), 4-(4,6-Dimethoxy-1,3,5-triazin-2-yl)-4-methylmorpholinium chloride (DMTMM), and trifluoroacetic acid (TFA) were purchased from Tokyo Chemical Industry Co., Ltd. (TCI) (Tokyo, Japan). Potassium iodide, benzotriazol-1-yloxy-tri(pyrrolidino)phosphonium hexafluorophosphate (PyBOP), 4,4′-azobis(4-cyanovaleric acid) (ABCVA), acetonitrile (super dehydrated), potassium hydroxide, hydrochloric acid (HCl), and formaldehyde solution (35%) were purchased from FUJIFILM Wako Pure Chemical Co. (Osaka, Japan). 1-Ethylpiperidine hypophosphite (EPHP) was purchased from Sigma (Marlborough, MA, USA). N-(2-hydroxypropyl) methacrylamide was purchased from Polysciences, Inc. (Warrington, PA, USA). Other reagents used in the experiments were purchased from Sigma-Aldrich, TCI, and FUJIFILM Wako Pure Chemical Co. 1,2-distearoyl-sn-glycero-3-phosphocholine (DSPC), 1,2-distearoyl-sn-glycero-3-phosphoethanolamine-n-methoxy(polyethyleneglycol)-2000] (mPEG2000-DSPE), and 1,2-distearoyl-sn-glycero-3-phosphoethanolamine (DSPE) were purchased from NOF (Tokyo, Japan). Cholesterol of analytical grade was purchased from FUJIFILM Wako Pure Chemical Co. (Osaka, Japan).

### 2.2. Animals

Female BALB/c mice (8–10 weeks old) weighing 20–25 g were purchased from Kyudo (Saga, Japan). All mice were acclimatized for one week before further experiments. All animal experiments were evaluated and approved by the Animal and Ethics Review Committee of Kyushu University.

### 2.3. Proton Nuclear Magnetic Resonance (^1^H NMR) Analysis

The chemical structure of the obtained product of each synthesis was characterized by 400 MHz ^1^H NMR ECZ400S (JEOL, Tokyo, Japan). All measurements were performed at room temperature. 

### 2.4. Gel Permeation Chromatography (GPC) Analysis

The molecular weight and molar-mass distribution of polymers were characterized by a GPC instrument with a refractive index detector (Shimadzu, Tokyo, Japan) equipped with 3 gel columns (guard column, α-M, α-M, and α-3000) from Tosoh (Tokyo, Japan). Dimethylformamide (DMF) with 10 mM lithium bromide (LiBr) and 0.1 M phosphate buffer pH 7.4 were used as eluent with a flow rate of 0.5 mL/min at 40 °C. The measurement was calibrated with poly(methyl methacrylate) (PMMA) and polyethylene glycol (PEG) standards. The chromatograms of PCB and the PAMPI polymer are presented in Figure S6.

### 2.5. Synthesis of PAMPI-Modified Lipids

A. Synthesis of **1**

2-Ethyl-2-oxazine was synthesized following previous reports [12,13]. Poly(2-ethyl-2-oxazine) was synthesized by the cationic ring-opening polymerization of 2-ethyl-2-oxazine (2.74 × 10^−1^ mmol) using an ethyl 3-bromopropionate initiator (3.91 × 10^−3^ mmol) and potassium iodide (KI, 3.91 × 10^−3^ mmol) in anhydrous acetonitrile (27 mL). The synthesis was performed according to a previous report [14]. Finally, a KOH (5 M)/methanol solution was added to quench the reaction, yielding polymer **1** with carboxylic acid and hydroxyl groups at the terminals [14,15]. Yield: 75%. ^1^H NMR (400 MHz, CD_3_OD) δ (ppm) 3.39 (t, J = 6.9 Hz, 4H), 2.42–2.19 (m, 2H), 1.04–0.90 (m, 3H).

B. Synthesis of **2**

Polypropyleneimine **2** was synthesized by the acid hydrolysis of **1** in HCl according to previous reports [16,17]. Yield: 80%. ^1^H NMR (400 MHz, CD_3_OD) δ (ppm) 2.52 (t, J = 7.3 Hz, 1H), 1.61 (p, J = 7.5 Hz, 1H).

C. Synthesis of **3**

Product **3** was obtained by the methylation of **2** via the Eschweiler–Clarke reaction; **2** was reacted at 105 °C for 2 days in a mixed solution of formic acid (85%) and formaldehyde solution (35%) (**2**: formaldehyde solution: formic acid = 1: 5: 8 molar ratio). This reaction solution was removed under reduced pressure to obtain a highly viscous white transparent solid. This was neutralized with a large excess of concentrated hydrochloric acid (2-fold excess of the amine in the polymer). Thereafter, the aqueous layer was extracted three times with diethyl ether to remove the formic acid complex. The mixture was then neutralized with sodium hydroxide and extracted with chloroform, followed by drying over potassium carbonate. Yield: 60%. ^1^H NMR (400 MHz, CD_3_OD) δ (ppm) 2.28 (t, J = 7.8 Hz, 1H), 2.13 (s, 1H), 1.63–1.51 (m, 1H).

D. Synthesis of **4**

tert-Butyl iodoacetate was synthesized according to a previous report [18]. Poly(*N*-methyl-propyleneimine) (0.033 mmol) and tert-butyl iodoacetate (1.5 eq) were reacted in 20 mL of acetonitrile at 60 °C for 12 h. Finally, the crude product was purified by re-precipitation in diethyl ether and dried in vacuum overnight. Yield: 89%. ^1^H NMR (400 MHz, CD_3_OD) δ (ppm) 4.52 (s, 1H), 3.88 (s, 4H), 3.53 (s, 3H), 2.51 (s, 1H), 1.49 (s, 12H).

E. Synthesis of **5**

Product **4** (0.1 mmol) was dissolved in 4 mL methanol. Meanwhile, DSPE (2 eq) and *N*,*N*-diisopropylethylamine (DIEA, 2 eq) were dissolved in 20 mL of chloroform. Meanwhile, PyBOP (14 eq.) was dissolved in 1 mL of DMF. The solutions were mixed and reacted at 45 °C for 5 days in a methanol/chloroform/DMF solvent mixture. After the reaction was completed, the mixture underwent size-exclusion column chromatography using Sephadex G-25 to separate unreacted lipids, unreacted polymers, and polymer–lipid conjugates. Finally, the solution was removed under reduced pressure using a rotary evaporator to obtain a yellow solid. Next, deprotection was conducted in TFA and product **5** was obtained after re-precipitation against diethyl ether 3 times, followed by dialysis and lyophilization. Yield: 68.3%. ^1^H NMR (400 MHz, CDCl_3_) δ (ppm): δ 4.71 (s, 2H), 3.58 (s, 2H), 3.24 (s, 3H), 2.18 (s, 1H).

### 2.6. Synthesis of PCB-Modified Lipids

The PCB-modified lipids were prepared according to the synthesis method in Scheme S1. PCB was synthesized via RAFT polymerization following our previous method [19] (Scheme S1). Briefly, 2-(dodecylthiocarbonothioylthio)-2-methylpropionic acid was used as a chain transfer agent and ABCVA was used as an initiator. Polymerization was conducted in DMF at 70 °C for 24 h after 4 cycles of freeze–pump–thaw for deoxygenation. The product was obtained after re-precipitation with diethyl ether, followed by vacuum drying as a yellow powder. The trithiocarbonyl thio terminus was subsequently removed using EPHP and ABCVA according to the previous method in [20]. The polymer with a hydrogenated terminus was obtained as a white product after re-precipitation in diethyl ether, followed by vacuum drying. The molecular weight of PCB was determined based on the repeating unit peak (1.0–2.0 ppm) and chain transfer agent end group (0.8–1.0 ppm) in Figure S2 (*M_n_* = 5 k). PCB-modified lipids were synthesized similarly to the PAMPI-modified lipids. ^1^H NMR (400 MHz, CDCl_3_) δ (ppm) (Figure S3): 0.5–0.7, 0.8–1.2, 1.0–1.8, 1.8–2.3, 3.1–3.5, 3.3–3.6, 3.6–3.8, 3.8–4.0.

### 2.7. Preparation of Liposomes

Polymer-modified liposomes were prepared using the Bangham method [21], similarly to a previous report [19]. The modification ratio of the polymer-modified lipid in the prepared liposomes was 5 mol% (PEG–liposome) or 10 mol% (PCB and PAMPI–liposome), and the lipid composition was DSPC/Chol/main-chain PCB-modified lipid = 2−x:1.0:x (x = 0.15 or 0.3) (molar ratio). Briefly, chloroform solutions of DSPC and Chol, as well as 2,2-trifluoroethanol and the polymer-modified lipid were mixed. The solvent was removed under reduced pressure by a rotary evaporator to form a lipid thin film. After vacuum-drying overnight, phosphate-buffered saline (PBS) was added to hydrate the lipid film at 65 °C. Next, the liposomes were passed through polycarbonate filters (400 nm, 200 nm, 100 nm, and 50 nm) using an extruder to adjust the size. The particle size and z-potential of the prepared liposomes were measured by a Zetasizer Pro (Malvern Panalytical, Malvern, UK) at 25 °C, and are summarized in Table 1.

### 2.8. Transmission Electron Microscope (TEM) Imaging

To confirm the size and morphology of the polymer-modified liposomes, TEM JEM-2010 (JEOL, Tokyo, Japan) was used to obtain an image of each liposome at an accelerated voltage of 120 kV. Liposome solution in ultrapure water (2 μL) was dropped onto copper grids and stained with 2 mg/mL gadolinium acetate for 2 min. The excess solutions were blotted with filter paper and the grids were dried at room temperature under reduced pressure.

### 2.9. Computational Method

PCB and PAMPI were constructed, and their charges were assigned by ACPYPE (AnteChamber PYthon Parser interfacE). Molecular dynamics (MD) simulations using a mechanical force field were completed using a Large-scale Atomic/Molecular Massively Parallel Simulator (LAMMPS) [22] based on Dreiding force fields. A single homo syndiotactic polymer with 20 degrees of polymerization and both ends of the chain terminated with hydrogens was modeled using molecular modeling software called Winmostar V10.00 [23]. For the homo polymer cell builder, chains were randomly placed in a cell with a three-dimensional periodic boundary condition. After building the polymer models, the initial configuration was created by placing about 2000 molecules of water around the model. First, 10 ps was performed at NVT (350 K), then 30 ps at NPT (1 atm, 300 k). After confirming that temperature, pressure, and density remained constant, the surrounding water was removed from the final MD structure, and the polymer structures were optimized by quantum chemical calculations (MNDO method) using the Gaussian 16 program package [24].

### 2.10. Calculation of Radius of Gyration and Scattering Function

In order to examine the difference in chain conformation between PCB, PAMPI, and also PEG in water, additional MD simulations were carried out. For the force field of the 10 mers of isotactic (i-) PCB, syndiotactic (s-) PCB, i-PAMPI, s-PAMPI, and PEG, the General AMBER force field (GAFF) [25] was adopted with (modified) Restrained Electrostatic Potential (RESP) charges [26,27], evaluated by ab initio calculations at the Hartree–Fock/6-31G [25] level considering the solvent (water) effects based on the integral equation formalism polarizable continuum model (IEFPCM). As for the water model, the TIP4P2005 model [28] was adopted. The MD engine used was GROMACS 2024.2 [29,30,31,32,33,34,35,36]. A single polymer chain and water molecules were arranged, in an initial state, in a cubic simulation box of 5 nm per side, with a periodic boundary condition so that the density of the system was nearly equal to 1 g/cm^3^. After the energy minimization and equilibration (10 ns) of the system under the NPT condition (1 bar, 298 K) with the Berendesen baro- and thermostats [37], sampling (20 ns) under the NPT condition (1 bar, 298 K) with the Parrinello–Rahaman barostat [38] and Nosé–Hoover thermostat [39,40] was carried out. In the series of simulations, all the bonds of molecules were constrained to the corresponding equilibrium length, the van der Waals interactions between non-bonded atoms were cut off at 0.9 nm, and the electrostatic interactions were calculated by the particle-mesh Ewald summation (PME) method with a cut-off length of 0.9 nm [41,42]. The radius of gyration *R_g_* based on the molecular geometry (without mass or charge weighting) and scattering function P(k) (corresponding to the one obtained by small-angle neutron scatting measurements) as a function of the magnitude *k* of the scattering vector of the polymer chain were then calculated on the basis of the MD trajectory obtained.

### 2.11. Evaluation of Blood Retention of Liposomes

The evaluation of the blood retention of polymer-modified liposomes was performed using a protocol modified based on a previous report [19]. Polymer-modified liposomes labeled with 1 mol% 1,1′-dioctadecyl-3,3,3′,3′-tetramethylindocarbocyanine perchlorate, DiI (5 μmol phospholipids/kg), were intravenously administered into BALB/c mice (n = 3). At 0.25, 1, 4, 8, and 24 h after administration, blood was withdrawn by the submandibular blood collection method. Serum samples were collected by centrifugation (2000× *g*, 15 min) after the blood was left for 15 min at room temperature. The fluorescent intensity of the DiI-labeled liposomes in the serum samples was measured using a fluorescence plate reader (λ_excitation_: 545 nm, λ_emission_: 585 nm).

### 2.12. Evaluation of Biodistribution of Liposomes

Polymer-modified liposomes containing 100 µL of DiI were injected into mice via the intravenous (i.v.) route at a quantity of 1 µmol/mL at day 0 and day 5. Organ collection was performed 24 h post-second injection and the fluorescence of DiI remaining in the organ was detected using an IVIS imaging system (λ_excitation_/λ_emission_ = 535/DsRed).

### 2.13. Evaluation of Antibody Production by the Liposomes

To evaluate the antibody production of the polymer-modified liposomes, the polymer-modified liposomes (0.01 μmol phospholipids/kg) were intravenously administered to BALB/c mice. At days 0, 3, 5, 7, and 14, submandibular blood collection was performed. Serum samples were collected by centrifugation after the blood was left for 15 min at room temperature. Antibodies against polymers were detected using ELISA according to the following protocol [43,44]. Briefly, polymer-modified liposomes (0.1 mmol phospholipids/mL) were added into a 96-well plate and incubated at 4 °C overnight. The plate was washed 3× with PBS, followed by 1 h of blocking with blocking buffer (1% BSA/TBS, pH 8.0). After 3 rounds of repeated washing, mouse serum (1:100) was coated on the plate and incubated for 1 h at room temperature, and then washed 5 times with PBS. HRP-labeled anti-mouse IgM (1:10,000) was then added to the plate and incubated for a further 1 h, followed by 5 rounds of washing. TMB substrate was added to the wells and incubated for 10 min. The color development was quenched by adding 2 M sulfuric acid solution. The absorbance of each well was detected at 450 nm using a plate reader.

### 2.14. Evaluation of Serum Protein Adsorption on Liposomes

The concentration of each polymer-modified liposome was adjusted (liposome, 10 mM, 10 mL + serum, 400 mL), followed by serum incubation at 37 °C for 15 min. The mixture was quenched on ice for 5 min. The liposomes with bound serum protein were collected by flowing the mixture to the size-exclusion chromatography (SEC) column (Sepharose 4 Fast Flow) with HBS buffer as the elution buffer (1.25 mL/min). The fraction was collected in an amount of 1400 mL. The fraction containing serum-bound liposomes was then concentrated using an Amicon^®^ Ultra Centrifugal Filter with 100 kDa MWCO; 500 mL liposome was added to the upper layer and centrifuged at 15,000× *g* for 5 min. The lower layer was removed and 300 mL liposome was added to the upper layer, followed by centrifugation using the determined speed and time. This procedure was repeated 3 times. The phospholipid (PL) concentration of the liposomes was evaluated by the acid digestion method based on the quantification of phosphorus content [45]. The PL concentration of each liposome was adjusted to a concentration of 2 mM in HBS buffer. The lipid content of the liposomes was disrupted by incubation of the liposomes in 10% SDS (liposomes: 10% SDS *v*/*v* = 1:1) at 95 °C for 5 min. Then, the reaction was quenched on ice for 5 min. A portion of this solution was taken for gel electrophoresis (10–20% gradient polyacrylamide gel, 220 V, reducing condition) to identify the proteins bound to the liposome, according to [46]. The bound protein was visualized by a Pierce™ Silver Stain Kit from Thermo Scientific™ (Waltham, MA, USA) according to the manufacturer’s protocol. The molecular weight (MW) of the proteins was visualized using the MagicMark™ XP Western Protein Standard (Invitrogen, Waltham, MA, USA).

### 2.15. Western Blot (WB) Assay of Bound C3 Protein

After the gel electrophoresis described above, the C3 complement protein was analyzed by immunoblotting. The protein samples after electrophoresis were transferred into the PVDF membrane and blocked with 5% milk in phosphate-buffered saline. The membrane was then incubated with polyclonal HRP-conjugated goat anti-mouse C3 antibody (GC3-90P-Z, Cosmo Bio, Tokyo, Japan) with 1:10,000 dilutions for 1 h at room temperature, followed by washing several times using PBS + 0.05% Tween 20. Protein bands were visualized by electrochemiluminescence at different exposure times. The MW of proteins was visualized using the ExcelBand All Blue Regular Range Protein Marker from SMOBIO Technology, Inc. (Hshinchu, Taiwan).

### 2.16. Statistical Data Analysis

All values are expressed as the mean ± SD. Statistical analysis was performed with a two-tailed unpaired *t*-test.

## 3. Results and Discussion

**Figure 2:**
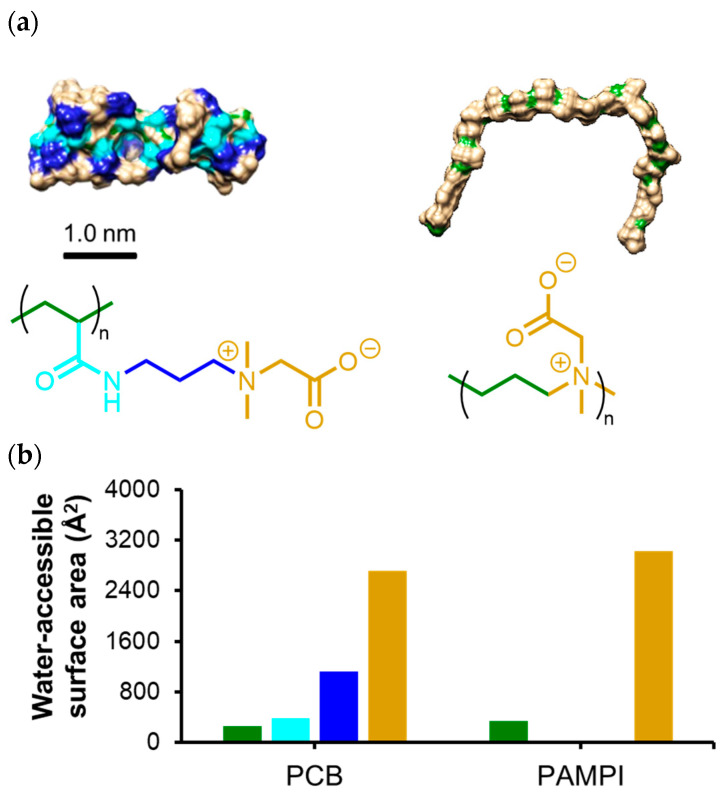
(**a**) Energy-minimized structure of PCB and PAMPI in water (**upper panels**) and chemical structures of polymers, showing the color of each group (**lower panels**). (**b**) Water-accessible surface area of each group of polymers.

The exposure of the component groups other than CB was evaluated from the energy-minimized structure of each polymer (PCB: syndiotactic 20-mer), as shown in Figure 2a. The water-accessible surface area of each group was determined, with the results summarized in Figure 2b. In PCB, the surface area of the CB group occupied 60% of the total surface area, and the other groups (propylene, amide, main-chain ethylene) occupied the remaining 40%. The exposure of propylene and amide was relatively large, which could be the epitope for antibody induction. In contrast, the surface area of PAMPI was almost solely occupied by the CB group (90%). This leads us to assume that PAMPI is superior to PCB in terms of its non-fouling nature against protein adsorption, leading to reduced antigenicity.

To compare the size of each polymer, the *R_g_* value was calculated for 10 mer of PCB and PAMPI with isotactic (i-) or syndiotactic (s-) stereochemical configurations, along with PEG (Table 2). In both cases of PCB and PAMPI, *R_g_* was independent of tacticity. PAMPI showed a somewhat (20—30%) larger *R_g_* value than that of PCB, irrespective of tacticity. This is consistent with the fact that while PCB has a bottle-brush-like compact conformation, having a thick cross-section (with a center located at the main-chain contour) because of its long side chains, PAMPI has an extended (semiflexible) conformation with a thin cross-section, as seen in Figure 2a. The value of *R_g_* for PEG is definitely smaller than the values for PCB and PAMPI, since PEG has a flexible (random-coil-like) conformation, as is well known. Such conformational behavior of these polymers can be confirmed from the Kratky plots of P(k) shown in Figure S4.

**Scheme 1:**
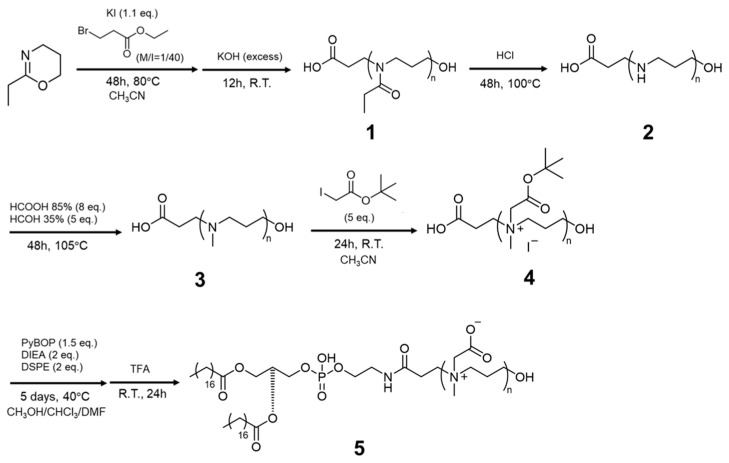
Synthesis of PAMPI-modified lipids.

PAMPI-modified lipids were synthesized to decorate the surfaces of liposomes following Scheme 1. Poly(2-ethyl-2-oxazine) was obtained by cationic ring-opening polymerization. The degree of polymerization was adjusted to be 40 (Table 3). This polymer was converted to poly(propylene imine) **2** via acid hydrolysis of the amide group. The *N*-monomethylation of **2** was conducted by reductive amination, using formaldehyde to obtain poly(*N*-methyl propyleneimine) **3**. Tert-butyl acetate was modified in the main-chain amine of **3** to give **4**. The ^1^H NMR analysis of **4** showed the quantitative modification of tert-butyl acetate in the main-chain amine group (Figure S1). PAMPI-modified lipids were prepared by the coupling reaction with DSPE, followed by the deprotection of tert-butyl ester.

**Figure 3:**
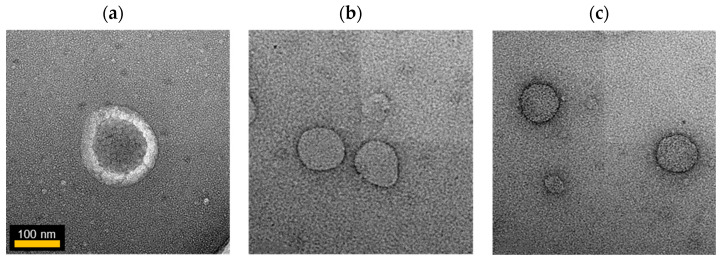
Representative TEM images of (**a**) PEG-, (**b**) PCB-, and (**c**) PAMPI-modified liposomes.

The polymer-modified liposomes were prepared as described in the Methods section. PCB-modified liposomes and PEG-modified liposomes were prepared as a control. The size, PDI, and ζ-potential of the liposomes are summarized in Table 1 and Figure S7. The size of the polymer-modified liposomes was controlled to be 80–90 nm (Figure 3). Polymer-modified liposomes are mono-disperse and have neutral ζ-potential.

**Figure 4:**
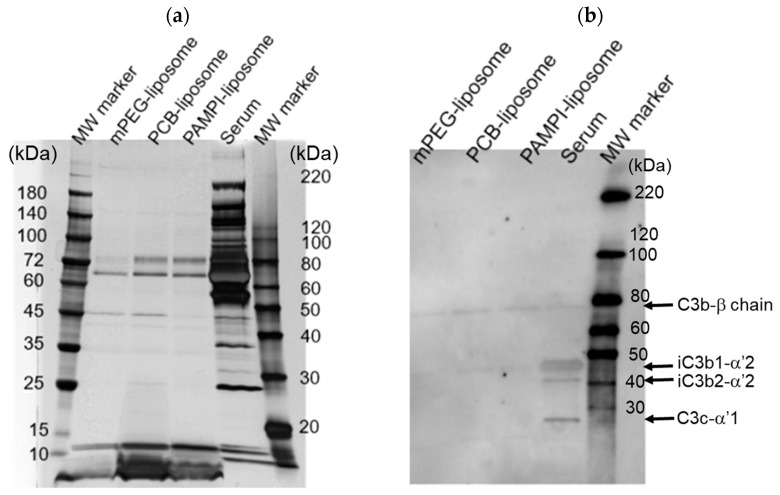
SDS-PAGE of serum protein adsorbed to polymer-modified liposomes visualized by silver staining (**a**), and Western blot of C3 protein adsorbed to polymer-modified liposomes (**b**).

The non-fouling nature of PAMPI-modified liposomes was compared with those of PCB- and PEG-modified liposomes based on serum protein adsorption. After treating the liposome with mouse sera, the adsorbed proteins were evaluated by PAGE with silver staining (Figure 4a). PAMPI- and PCB-modified liposomes showed a somewhat higher adsorption of serum proteins than PEG-modified liposomes. Bands of 73, 66.5, and 50 kDa were assignable to heavy-chain immunoglobulin, C3, and Apo A-VI, respectively [47]. The adsorption of complements to each liposome was confirmed by Western blotting (Figure 4b). PAMPI- and PCB-modified liposomes showed a weak adsorption of complements (C3b-*β* and iC3b1-*α*’2) as PEG-modified liposomes. Collectively, the non-fouling nature of PAMPI-modified liposomes was not inferior to PCB- and PEG-modified liposomes.

**Figure 5:**
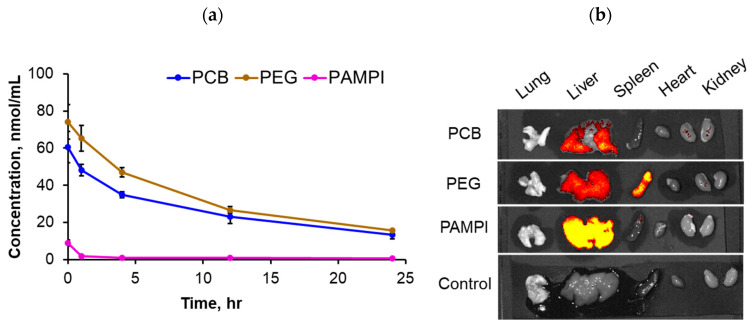
Blood retention profiles of polymer-modified liposomes after i.v. injection in mice (**a**) and representative image of biodistribution of polymer-modified liposomes 24 h post-first injection (**b**). Each value in (**a**) represents the mean ± S.D. (n = 3).

We examined the blood retention of the PAMPI-modified liposomes in mice after intravenous injection. PCB- and PEG-modified liposomes were also evaluated as comparisons. As shown in Figure 5a, the blood retention of PCB-modified liposomes was comparable to the PEG-modified liposomes, which is consistent with our previous report [19]. Unexpectedly, the blood retention of PAMPI-modified liposomes was much shorter than that found for the PCB-modified liposomes. PAMPI-modified liposomes immediately disappeared from the blood circulation within 1 h of injection, which is equivalent to naked liposomes (Figure S5). As shown in Figure 5b, the PAMPI-modified liposomes were particularly accumulated in the liver 24 h post-injection, and the same was found for PCB- and PEG-modified liposomes. It is unknown why PAMPI-modified liposomes showed very short blood retention, as their non-fouling nature did not seem to be inferior to PCB- and PEG-modified liposomes (Figure 4).

**Figure 6:**
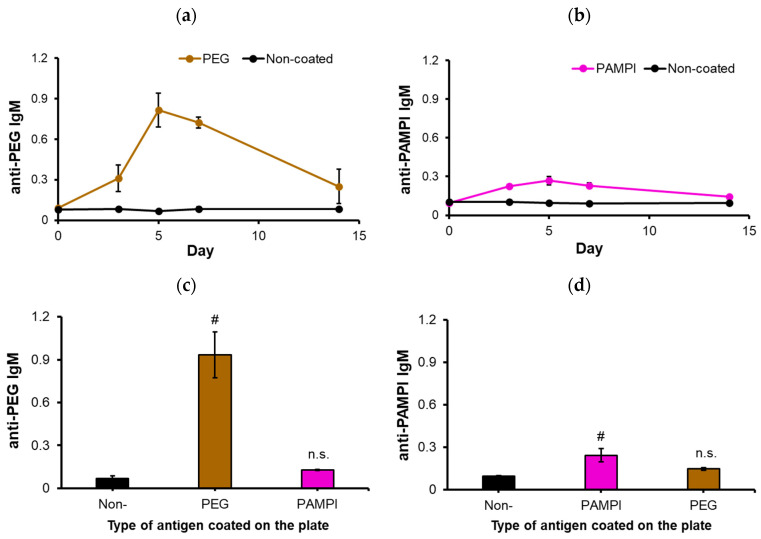
Time dependence of IgM titer induced by PEG- (**a**) and PAMPI-modified liposomes (**b**). The binding of anti-PEG IgM (**c**) and anti-PAMPI IgM (**d**) induced by corresponding liposomes to the polymer-coated plate. IgM against each polymer was detected by ELISA (**c**). Each value represents the mean ± S.D. (n = 3). *p* values apply to differences between anti-polymer IgM of the non-coated plate and polymer-coated plate (# *p* ≤ 0.05, n.s. *p* > 0.05).

The generation of anti-polymer IgM in serum after the intravenous injection of each liposome was evaluated by ELISA using a liposome-immobilized plate. Figure 6a shows the time-dependent change in anti-PEG IgM amount induced by PEG-modified liposomes. The amount of anti-PEG IgM reached a maximum at around day 5, which is consistent with our previous report [19]. Figure 6c shows the specificity of anti-PEG IgM induced by PEG-modified liposomes. Anti-PEG IgM is specifically bound to PEG over PAMPI. PAMPI-modified liposomes also induced anti-PAMPI IgM, but at a low amount compared with anti-PEG IgM (Figure 6b). The binding of anti-PAMPI IgM was not so specific to PAMPI (Figure 6d). It has been reported that polymer-modified liposomes with short retention times do not show antibody-induced rapid clearance in the second injection [48], showing negligible IgM induction toward such polymer-modified liposomes. Thus, the weak induction of anti-PAMPI IgM is due to the short blood retention time of liposomes.

## 4. Conclusions

We designed a novel PCB, PAMPI, composed of minimal components. The CB group was included as a potential epitope for antibody induction. PAMPI-modified liposomes were successfully prepared. To our surprise, PAMPI-modified liposomes showed very short blood retention compared to conventional PCB, although their non-fouling nature estimated by serum protein adsorption was not inferior to PEG- and PCB-modified liposomes. The short blood half-life of PAMPI is also partly explained by its semi-flexible conformation; meanwhile, PCB with compact conformation shows a longer blood half-life. As a result of this short blood retention, PAMPI-modified liposomes did not induce a significant amount of anti-PAMPI IgM. In conclusion, PAMPI is not a suitable polymer for extending the blood retention of liposomes, even though it has minimal components except for the CB group.

## Figures and Tables

**Table 1:** The properties of polymer-modified liposomes.

Properties	PCB	PAMPI	PEG
Modification ratio on liposome (mol%)	10	10	5
Liposome diameter (nm)	78 ± 0.39	87 ± 0.34	81 ± 1.21
Polydispersity index (PDI) of liposome	0.037 ± 0.01	0.031 ± 0.02	0.002 ± 0.02
ζ-potential (mV)	−8.3 ± 1.97	−2.1 ± 1.74	−2.4 ± 0.03

**Table 2:** The calculated and reported *R_g_* values of each polymer with 10 mer.

Type of Polymer	Tacticity	*R_g_* (nm)
PCB	Syndiotactic	0.946
	Isotactic	0.940
PAMPI	Syndiotactic	1.14
	Isotactic	1.19
PEG	N/A	0.603

**Table 3:** The molecular weight and molecular weight distribution of each polymer, as determined by GPC.

Properties	PCB	PAMPI	PEG
Molecular weight (*M_n_*)	6424 ^A^	19,223 ^B^	5000 ^C^
Molecular weight distribution (*M_w_*/*M_n_*)	1.08 ^A^	1.44 ^B^	N/A
Degree of polymerization	20	40	45

^A^ Determined by GPC of compound **1** relative to PEG standard. ^B^ Determined by GPC relative to PMMA standard. ^C^ PEG is available as a commercial product.

## Data Availability

The raw/processed data required to reproduce these findings are available upon request.

## Supplementary Materials

The following supporting information can be downloaded at: https://www.mdpi.com/article/10.3390/pharmaceutics16101271/s1, Figure S1: ^1^H NMR spectra of compounds **1** to **5**; Scheme S1: Synthesis of PCB-modified lipids; Figure S2: The ^1^H NMR spectra of PCB (400 MHz, CDCl_3_); Figure S3: The ^1^H NMR spectra of PCB-modified lipids (400 MHz, D_2_O); Figure S4: Polymer conformational behavior represented by Kratky plots of P(k); Figure S5: Blood retention profile of PAMPI-modified liposomes vs. naked liposomes. Figure S6: The GPC chromatogram of (a) PAMPI and (b) PCB; Figure S7: The size-distribution curve of each polymer-modified liposome characterized by DLS; Figure S8: The properties of naked liposomes assessed in Figure S5 characterized by DLS; Figure S9. The biodistribution of polymer-modified liposomes (n = 3) detected at 24 h post-second administration of liposome.
