# Supplementary material for: A Stealthiness Evaluation of Main Chain Carboxybetaine Polymer Modified into Liposome
Source: Pharmaceutics. 2024 Sep 28;16(10):1271. doi: 10.3390/pharmaceutics16101271 (PMC11510557; doi:10.3390/pharmaceutics16101271)
Supplement: Supplementary file 1 [file pharmaceutics-16-01271-s001.zip › pharmaceutics-3198883-supplementary.pdf]

Supplementary Information

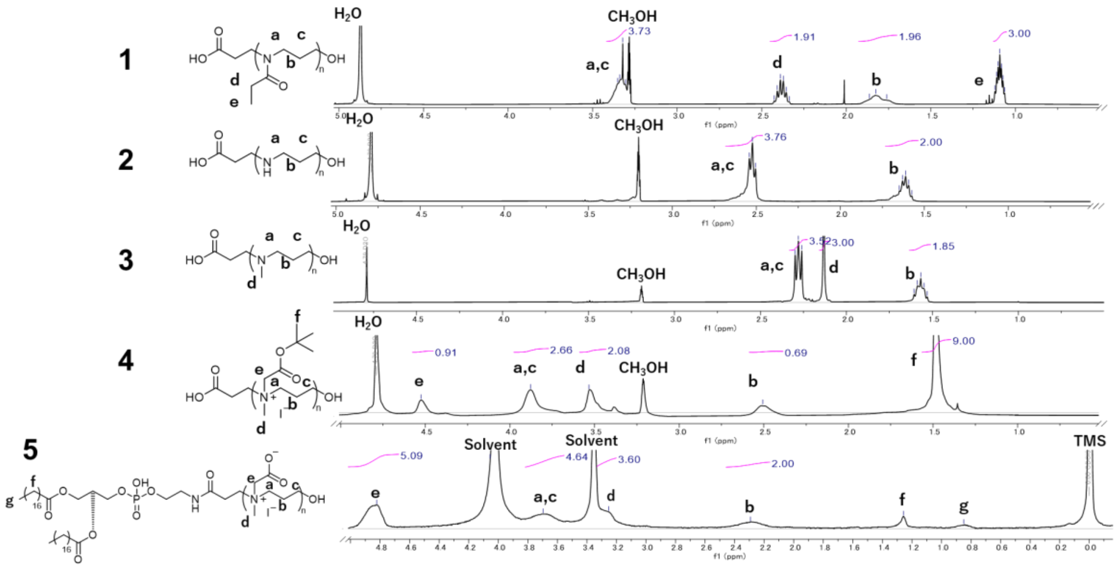

Figure S1. The ^{1}H NMR spectra of compound **1** to **5.**

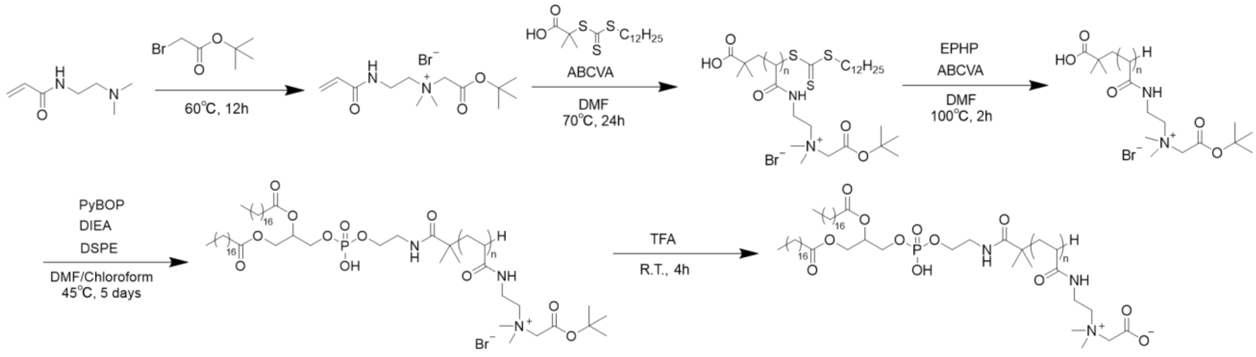

Scheme S1. Synthesis of PCB-modified lipid.

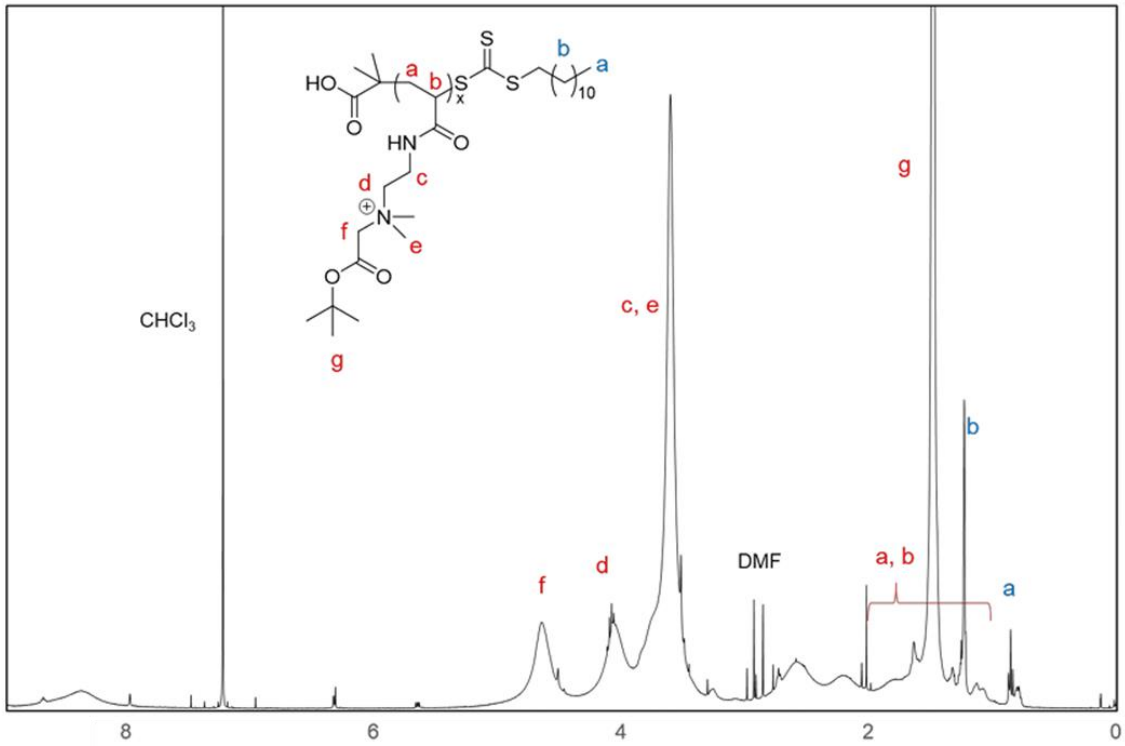

Figure S2. The ^{1}H NMR spectra of PCB (400 MHz, $CDCl_3$).

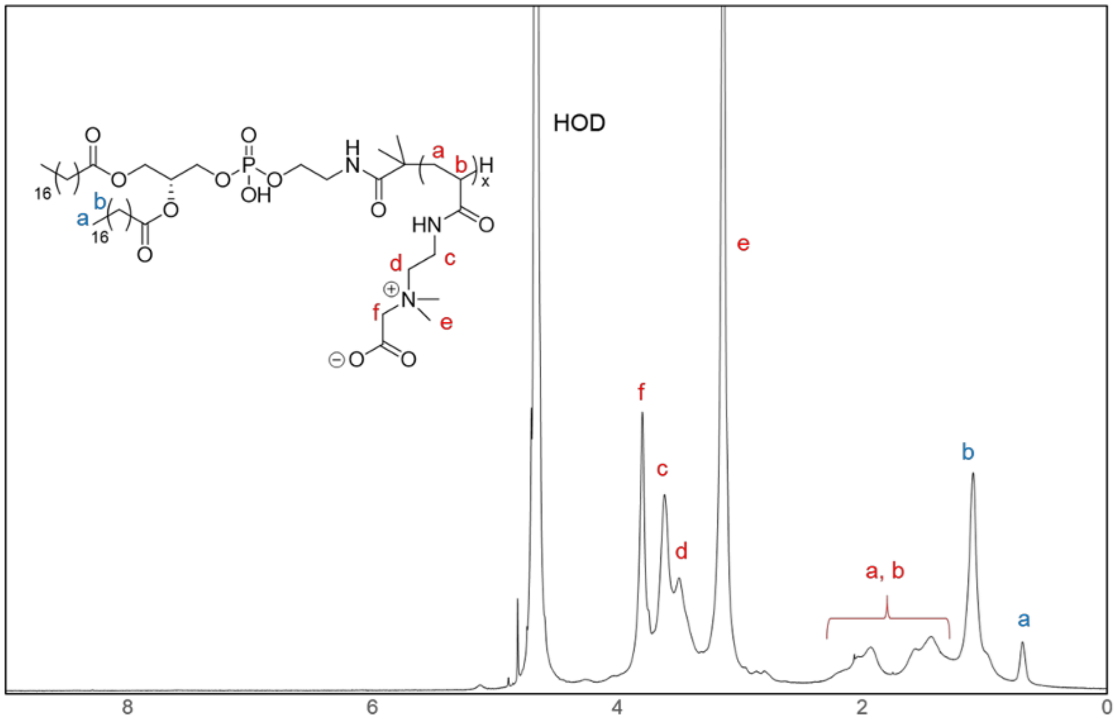

Figure S3. The ^{1}H NMR spectra of PCB-modified lipid (400 MHz, D_2O).

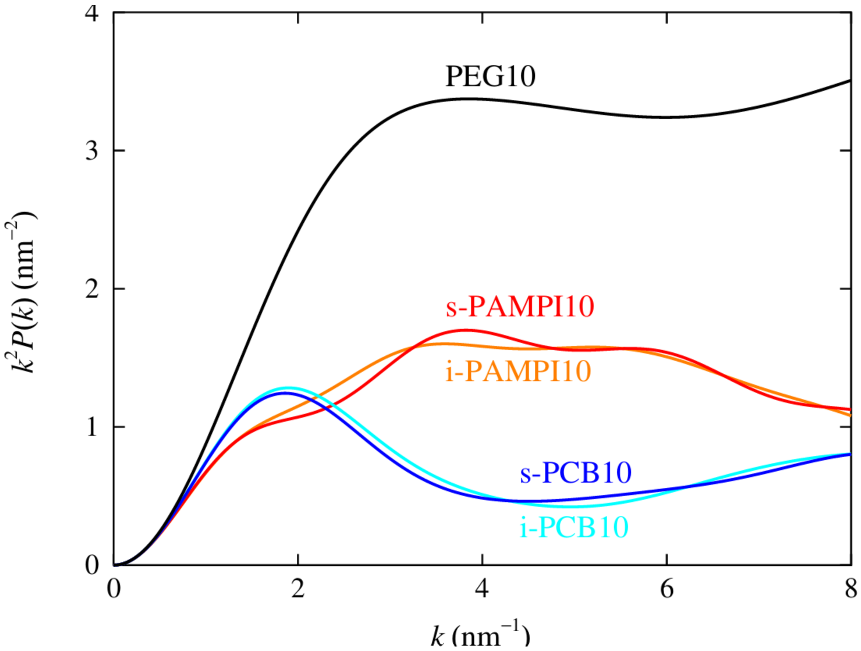

Figure S4. Polymer conformational behavior represented by Kratky plots of P(k).

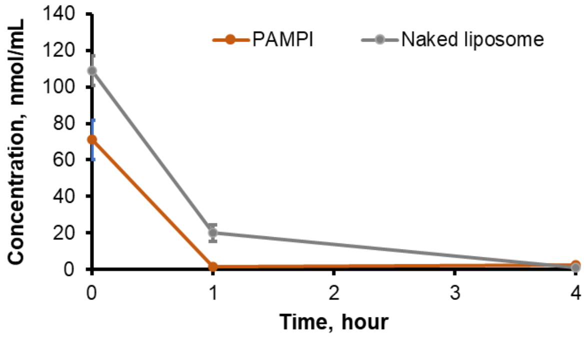

Figure S5. Blood retention profile of PAMPI-modified liposome vs naked liposome within 4h post-injection. Each value represents the mean ± S.D. (n = 3).

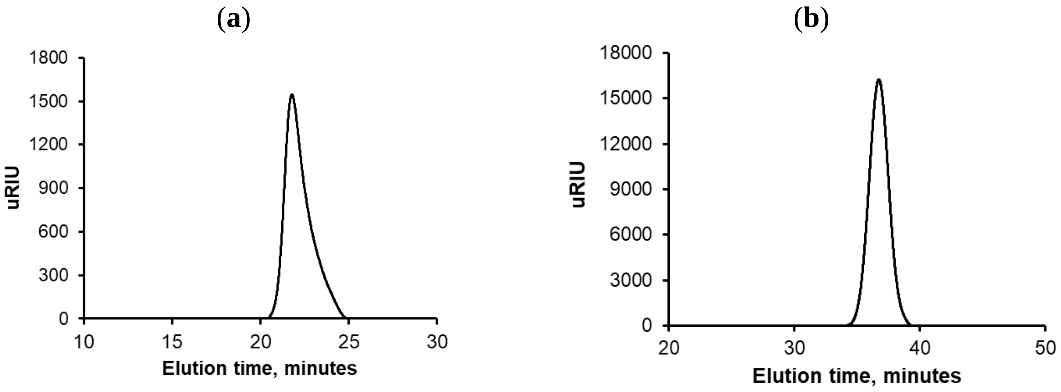

Figure S6. The GPC chromatogram of (a) PAMPI and (b) PCB.

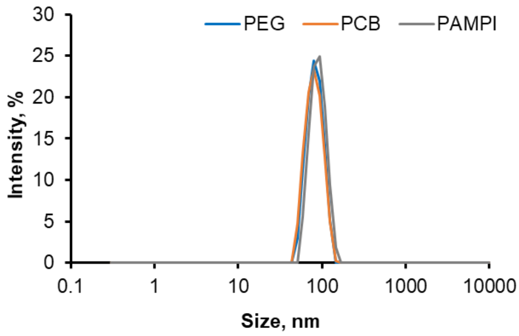

Figure S7. The size-distribution curve of each polymer-modified liposomes characterized by DLS.

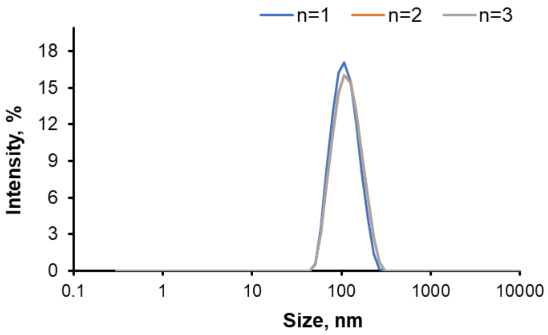

Diameter, nm	108.53 ± 2.27
PDI	0.13 ± 0.01
ζ–potential, mV	-8.3 ± 6.3

Figure S8. The properties of naked liposome assessed in Figure **S5** characterized by DLS.

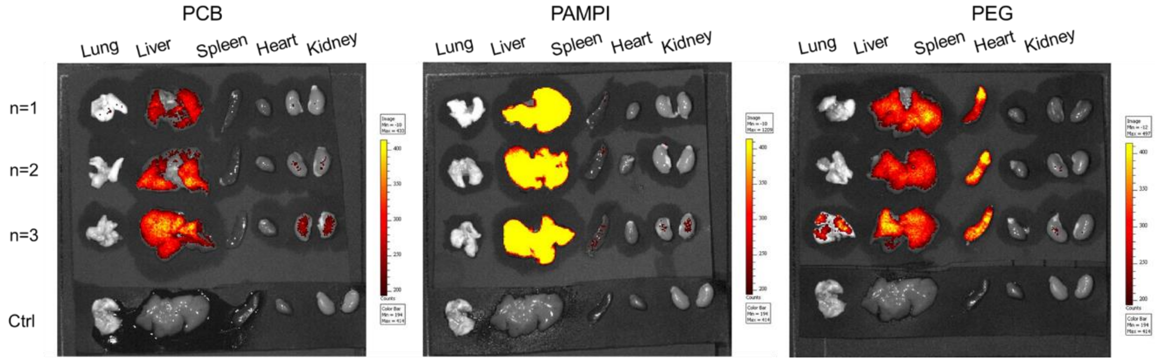

Figure S9. The biodistribution of polymer-modified liposome (n= 3) detected at 24h post-second administration of liposome.
